# Supplementary material for: An automated morphometric approach to evaluate distal lung patterning in mouse models of bronchopulmonary dysplasia
Source: PLoS One. 2026 Feb 17;21(2):e0333159. doi: 10.1371/journal.pone.0333159 (PMC12912560; doi:10.1371/journal.pone.0333159)
Supplement: Attachment — Submitted filename: Response to Reviews.docx [file pone.0333159.s007.docx]

**Response to Reviewer Comments:**

Reviewer #1:

The work by Gainer and colleagues described a novel method to examine the distal patterning of the lung applied to models of bronchopulmonary dysplasia. The authors convey the importance of using unbiased methods to measure the distal lung architecture in lung disease. The manuscript describes a semi-automated method for quantifying alveolar ducts and alveoli using BPD as a model to test the tool. The work will be of interest to the journal's readership, particularly researchers in pulmonary biology. I am supportive of the manuscript's acceptance; however, the authors should address the following issues.

C1. Authors should elaborate on whether the described method has the potential to be utilized independently or even replace standard measurements, such as the manual linear intercept.

R1. This is an excellent suggestion that we have expanded upon in Paragraph 4 of the Discussion. Our primary recommendation would be to use this method to supplement measures such as L_M_ and RAC, which do not explicitly discriminate between subdivisions of respiratory airways. This application alone is not problematic, nor does it invalidate these measures, but instead provides an opportunity for the derivation of additional information by describing whether large or small respiratory airways are altered by disease in tandem, or whether changes are specific to one type of airway that is driving changes in L_M_ or RAC. We note that the current tool is limited by the same factors that may influence L_M_ and RAC, such as unequal lung processing, biased image acquisition and improper stereology. While our macro could be utilized to assess lung structure independently of these standard measurements, it is not intended to replace these measures.

C2. While the validation using the BPD model is critical to validate the model, could the researchers perform additional measurements utilizing other disease models in older mice?

R2. This is an excellent suggestion by the reviewer, and would be advantageous in demonstrating the generalizability of this macro to other disease models, such as emphysema or chronic bronchitis, both of which fall under the umbrella of COPD. However, we do not have access to any tissues that we could use for the purposes of validation in a separate model, nor are there any laboratories at Queen’s University that specialize in mouse models of such diseases, limiting opportunities to do so.

C3. Could the macro be utilized or adapted to use with other image processing software?

R3. This is an excellent question. The macro was initially written in ImageJ Macro Language (IJM) and utilizes two packages that are native to or were developed for FIJI. As such, it was designed to be used exclusively with FIJI. While it is possible that this macro could be adapted for use with other open-source image processing programs, such as QUPath, there are several factors that would make this relatively difficult. The first of such challenges would be syntactical differences between scripting languages. For example, QUPath utilizes Groovy, a separate scripting language, while our macro is written in IJM, which is native to FIJI. These two languages are dissimilar, which would necessitate a complete rewrite/refactoring. Moreover, the packages that this macro utilizes, including FIJI’s particle analyzer and an excel sheets toolkit, have no direct equivalent in QUPath (an issue that likely extends to other programs), Further development would be needed to create equivalent functions in the target software’s scripting language. Our group is not familiar with Groovy, and we do not routinely utilize software packages outside of QUPath and FIJI, so this adaptation would be difficult. Given that the macro’s structure isn’t complex, however, individuals who are proficient in both Groovy and IJM would most likely be able to adapt it for different software. It is also important to note that FIJI is broadly used and freely accessible throughout the scientific community, making it an ideal platform for this analysis tool.

C4. Figure legend 1 is somewhat confused. For example, "(D) binarized version of D", or perhaps the binarized version of C. Please clarify.

R4. Thank you for this observation – we have corrected the figure legend to reflect the correct panels.

Reviewer #2:

This manuscript describes the development of a FIJI script to substantially automate the morphometric analysis of distal lung patterns in mammals. The semi-automated script reduces both hands-on time to make the measurements and possible operator bias. The developed script was benchmarked against the existing methods of mean linear intercept (LM) and was found to compare favorably.

The methods, statistics and analysis in the paper appear to be appropriate. The detailed statistical tests exceed this reviewer’s knowledge of statistics, but visual inspection of the processed and raw data suggests that the method is accurate and very useful. The text is generally clear and the diagrams are generally good, although specific suggestions to improve the text and figures are included below.

Overall this paper is appropriate to publish in PLOS ONE and should significantly benefit pulmonary research.

C1. In Figure 1 H and I, there is a considerable amount of tissue that is non marked off as non-targeted structures, and is neither large airways nor alveoli. (i.e. if you merge H and I, what is all the unmarked white space?). I don’t think this is described in the text, but I think there should be some explanation of the uncategorized tissue and some quantification of it. I would guess that at least 20% of the image is unaccounted-for space. Marking an example in Fig. 1I and explaining it in the text or legend would help a lot.

R1. This is a valid concern that the initial submission did not discuss in great detail. We had felt during development that it was preferable to exclude potentially ambiguous airways from the analysis, as a means of avoiding misclassification. As such, we limited our analyses to the airways that were visually classifiable as either alveoli or alveolar ducts. We have now included an additional figure in the revised manuscript, **Fig. 5**, which demonstrates that these intermediate airways account for 30-40% of the total quantified airspace in the validation dataset. Although this value is relatively large, we demonstrate that this percentage is significantly reduced by hyperoxia, alongside a reduced number of these airspaces per FOV. This observation, which is discussed in the final page of the revised Results and page 2 of the revised Discussion, is consistent with the notion of impaired branching in our disease model.

C2. line 48 and elsewhere: The authors report their results show that hyperoxia-exposed mice possess fewer, enlarged alveoli that occupy less total area as well as enlarged alveolar ducts that occupy more space. How much of this was known before (which make for good validation of the technique) and how much is new results? If know, references are in order. If new results, a phase statement something along the lines of “ …. , effects that were not previously known.”

R2. The reviewer poses an excellent question. BPD is, phenotypically, a well-characterized disease, in which both enlargements alveolar ducts and alveoli have been described in both humans in mice. We have updated the manuscript to include three new references which describe alveolar duct and alveolar enlargement in both humans as mice. These citations support our findings as measures of model validation, rather than a novel discovery that is linked to disease development. These references are included on lines 330 and 340 of the revised manuscript.

C3. line 53: How much faster is using the pipeline than doing a manual LM? The manuscript claims improved processing times but does not quantify the improvements.

R3. To address this important point, we measured the time required to process one set of images (*n* = 6) from an optimization animal, either manually or using our macro. We demonstrate that manual measurements, involving the application of test limes to images and subsequent quantification of intercepts, requires approximately 52 seconds per image (52.23 ± 5.169). In comparison, quantification of airspace characteristics with our macro requires approximately 14 seconds (14.30 ± 2.855) per image, demonstrating a substantial (~3.5X) improvement over manual quantification. This time savings represents a benefit that is over and above the additional structural information provided by our macro. These results have been included in **Fig. 2K**. It is important to note that these measurements were collected on a MacBook Pro with an M1 Max processor and 32 GB RAM, and experienced processing times may vary depending on computational power, though this macro is not resource-intensive.

C4. line 162. Why were a subset of mice given a subcutaneous dose of 5 ul of PBS prior to hyperoxic exposure. First, why was this done? There does not seem to be a treatment involving injection of a drug, so why inject such a small amount of PBS below the skin? Second, how was 5 ul of PBS reliably delivered? 5 ul in a microfuge tube is not hard, but 5ul subcutaneously seems technically challenging. Third, where was the mouse injected? Fourth, I couldn’t find a sub-group of injected broken out as a separate data set? Was this group pooled with the non-injected or was the data not used? If not used, please remove this description from methods.

R4. The 5 µL PBS injection was only administered to the mice sacrificed at P7, which were used to validate the method. Mice in both the normoxia and hyperoxia groups were equally treated, as they had initially served as vehicle controls in a separate study examining the effects of an experimental therapeutic on lung development in an acute mouse model of BPD. In an effort to adhere to Russel and Burch’s principle of the Three Rs, as outlined by the Queen’s University CCAC, we sought to reduce our usage of mice by reusing tissues from the aforementioned experiment, rather than sacrificing several additional litters of pups to perform identical endpoint procedures and analyses. The 5 µL PBS injection was administered to pups at postnatal day 0 (P0) using a 5 µL Hamilton Syringe. The injection was directed into the skin-fold present above the interscapular region. As the P7 pups were the only group that received S.Q. injections, no sub-groupings were performed. We have amended the description in the methods to clarify that the P7 subset, not the P14 subset, received this injection.

C5. lines 231 and 234 Tables 1 and 2. This needs a legend, and I think it would be less confusing if “Circularity” were replaced with “Circularity values tested”. There needs to some explaination for why there are two or three values in each cell on the Circularity row.

R5. Thank you for the suggestion. We have replaced “Circularity” with “Circularity values tested”. The presence of 2-3 values in the “Circularity” rows was meant to reflect that each of those circularity values was tested, while limiting area to the corresponding value in the “Airspace Area” row, This point was poorly communicated in the original manuscript. We have modified the “Method optimization” section to elaborate on the values in Tables 1 and 2.

C6. Figure 1. Lots of thing should be explicitly pointed out for the reader.

a. In C, there should be arrows or something indicating the manually removed nontarget structures. Making the black mark offs a bright orange would help make them visible.

R6a. This is a great suggestion. We have revised the coloring scheme for manually removed nontarget structures, making these structures orange. We have also altered the outputs so that all detected airways are cyan. These changes have been reflected in all figures. The methods section has also been amended to describe these modifications.

b. in D and E, I could not actually find an example of a disconnected exudate or debris that had been removed. Please indicate in D and/or E examples of what changed.
c. in F/G I couldn’t find examples of small spaces that had been filled. If they are too small to see in the figure, include insets to show the effect.

R6b/c. For **Fig. 1D/E/F/G**, we have included insets to better demonstrate the effects of the binary operations.

d. Per the concern above, in H and I, there are still many spaces that are not colored in H or I. Why are these very nice looking spaces not colored and what percent of the total area do such spaces occupy? Please explain in the text or the legend.

R6d. This is a valid observation. We avoided coloring these airspaces for two principal reasons. The first of these reasons, as discussed in R1, as to exclude potentially ambiguous airways from the analysis. Secondly, we felt that having too many colored spaces on the output image would result in visual clutter, in turn detracting from the contrast the coloring aims to provide. Although the changes made in **R6a** may mitigate this cluttering, it would require additional operations to color these intermediate airspaces. We felt that such a change would increase the complexity and computation time for the macro, while providing little benefit, and as such included the option to quantify these intermediate airspaces as a separate operation in the macro. We have added a brief description to the caption in **Fig. 1** describing these excluded airways. We have also performed a quantification of these airways in our validation dataset (included as **Fig. 5**), as described in R1.

C7. Fig. 2 There are blue and orange circles is no key in the figure and no explanation in the legend of what these colors mean. Please add keys such as those in Fig. 3G or fig. 4E.

R7. We have included a visual key in **Fig. 2** in order to convey that blue dots represent control (21% O_2_) mice and orange dots represent BPD (95% O_2_) mice.

C8. Fig. 2 The red dots overlapping with the blue dots in D are very hard to see. Can those be made a little bigger?

R8. The stroke width of the red dots was increased from 0 -> 2 point, and the stroke width of the black dots was increased from 0 -> 0.5 point. With both dots increased in size, but the red dots increased more substantially, both points should be easier to discern, even when overlaid.
